# Osseointegration of a New, Ultrahydrophilic and Nanostructured Dental Implant Surface: A Comparative In Vivo Study

**DOI:** 10.3390/biomedicines10050943

**Published:** 2022-04-19

**Authors:** Andreas Pabst, Ashraf Asran, Steffen Lüers, Markus Laub, Christopher Holfeld, Victor Palarie, Daniel G. E. Thiem, Philipp Becker, Amely Hartmann, Diana Heimes, Bilal Al-Nawas, Peer W. Kämmerer

**Affiliations:** 1 Department of Oral and Maxillofacial Surgery, Federal Armed Forces Hospital, Rübenacherstr. 170, 56072 Koblenz, Germany; andipabst@me.com (A.P.); becker-ph@web.de (P.B.); 2 Department of Oral and Maxillofacial Surgery—Plastic Operations, University Medical Center Mainz, Augustusplatz 2, 55131 Mainz, Germany; cholfeld@students.uni-mainz.de (C.H.); daniel.thiem@uni-mainz.de (D.G.E.T.); amelyhartmann@web.de (A.H.); diana.heimes@unimedizin-mainz.de (D.H.); al-nawas@uni-mainz.de (B.A.-N.); 3 Morphoplant GmbH, Universitätsstr. 136, 44799 Bochum, Germany; ashraf.asran@morphoplant.de (A.A.); luers@morphoplant.de (S.L.); laub@morphoplant.de (M.L.); 4 Laboratory of Tissue Engineering and Cellular Culture, State University of Medicine and Pharmaceutics “Nicolae Testemitanu”, Stefan cel Mare si Sfant Boulevard 165, 2004 Chisinau, Moldova; vpalarie@gmail.com

**Keywords:** implant, osseointegration, acid-etched, sand-blasted, micro- and nanostructured, ultrahydrophilic

## Abstract

This study compared the osseointegration of acid-etched, ultrahydrophilic, micro- and nanostructured implant surfaces (ANU) with non-ultra-hydrophilic, microstructured (SA) and non-ultrahydrophilic, micro- and nanostructured implant surfaces (AN) in vivo. Fifty-four implants (*n* = 18 per group) were bilaterally inserted into the proximal tibia of New Zealand rabbits (*n* = 27). After 1, 2, and 4 weeks, bone-implant contact (BIC, %) in the cortical (cBIC) and spongious bone (sBIC), bone chamber ingrowth (BChI, %), and the supra-crestal, subperiosteal amount of newly formed bone, called percentage of linear bone fill (PLF, %), were analyzed. After one week, cBIC was significantly higher for AN and ANU when compared to SA (*p* = 0.01 and *p* = 0.005). PLF was significantly increased for ANU when compared to AN and SA (*p* = 0.022 and *p* = 0.025). After 2 weeks, cBIC was significantly higher in SA when compared to AN (*p* = 0.039) and after 4 weeks, no significant differences in any of the measured parameters were found anymore. Ultrahydrophilic implants initially improved osseointegration when compared to their non-ultrahydrophilic counterparts. In accordance, ultrahydrophilic implants might be appropriate in cases with a necessity for an accelerated and improved osseointegration, such as in critical size alveolar defects or an affected bone turnover.

## 1. Introduction

Implant-supported oral and dental regeneration and rehabilitation can be a valid second chance in cases where natural teeth are compromised and/or not available for a tooth-fixed prosthesis [1]. It is known, that dental implants allow a secure and reliable fixation in toothless cases and can therefore significantly improve the prosthetic function, the aesthetic appearance and patients’ overall satisfaction with the treatment outcome. Interestingly, comparing prosthetic restorations on dental implants and on natural teeth, there seems to be no difference concerning patients’ satisfaction [2]. Overall, dental implants represent an irreplaceable treatment option in oral regeneration with a continuous improvement concerning aesthetics, functionality, and long-term results [3,4]. There are convincing long-term implant survival and success rates that may exceed 94% and higher even after 10 years of follow-up [5,6,7]. In this context, oral hygiene has been identified as one of the most relevant factors influencing the risk for implant failure and overall implant survival and success rates. Exemplarily, Cheung et al. demonstrated that a deficit concerning interproximal purification and an uncontrolled and increased plaque accumulation can cause peri-implant diseases [8], such as periimplantitis that can be associated with an increased risk of implant failure [9]. Next to implant failure, oral hygiene was even reported to be a relevant aspect concerning the performance of prosthesis on natural teeth and dental implants [10]. The relevance of oral hygiene in implant dentistry is even addressed by the development of antimicrobial implant surface coatings, such as different nano coatings, in order to reduce bacterial contamination on implant surfaces [11]. Further, there exists a direct association between oral health and hygiene and quality of life [12].

In general, patients’ expectations have been rising and they are increasingly looking for comfort in terms of immediate implant placement and loading as well as shorter healing times [13,14,15,16]. Also, patients with conditions and/or diseases influencing bone turnover wish to be regenerated by implant-supported restorations [17]. In those compromised patients, implant survival and success rates might be reduced, with complication rates increased [3,18,19,20]. Therefore, future challenges are immediate implant placement and loading in patients with non-degenerative conditions and implant placement in compromised patients [16,21]. This could be achieved through improvement of the implants’ osseointegration, which is considered as a structural and functional connection between bone and implant [22].

Implant design can be divided into macro-, micro-, and nanostructure as well as chemical composition and physical parameters [21,23]. Next to the macro- microstructure design, surface modifications and coatings are of interest. These can be divided into physical, chemical, and biological/biomimetic ones. Here, a multitude of different surface treatments such as carbon, bisphosphonate, growth factor, hydroxyapatite, and calcium phosphate coatings, among some others, were reported [17,24,25]. Unfortunately, none of the growth factor coatings are routinely used for clinical application for several reasons, such as biological instability and due to legal regulations. In this context, chemical and micro-structured modifications might be more promising. This might include sandblasting and acid-etching. Next, a modification of the wettability and (ultra-) hydrophilicity can be relevant to improve the biological performance and osseointegration [21,26,27,28]. With respect to osseointegration, Ríos-Santos et al. analyzed and demonstrated in an animal vivo experiment that the extent of osseointegration can be significantly influenced via implant design. Consequently, the macro- and microscopic implant design has to be addressed in the course of developing new and innovative dental implant systems [29]. With a special focus on narrow diameter dental implants and therefore the macroscopic implant design, Asaaf et al. retrospectively analyzed narrow diameter dental implants with sufficient implant success (89%) and cumulative survival rates (99%) up to 9 years of follow-up. Interestingly, this study demonstrated that there was no correlation between the design of the prosthetic restoration and overall implant complication rates, neither for biological nor biomechanical complications [30]. Further, osseointegration can be directly or indirectly influenced by patient-specific factors, such as bruxism and temporomandibular disorders. In these cases, the use of an occlusal splint can be useful to decrease the powerful loads caused by bruxism and temporomandibular disorders that can appear on dental implants and its prosthetic superstructure. Occlusal splints can preserve prosthetic restorations and can further influence osseointegration of dental implants, e.g., following immediate loading protocols [31].

Next to macro- and microscopic implant and surface characteristics, alternative instruments for implant site preparation and implant placement are of relevance for implant placement. In this context, Bennardo et al. performed a systematic review analyzing the utilization of magnetic mallets for dental implant placement. The findings of this study demonstrated that dental implants placed in the experimental group showed an increased survival rate compared to the control group (99% vs. 95%) [32]. Also, primary implant stability can even be influenced by surgical methods and by osseodensification techniques representing a fundamental aspect for implant success. In this context, Inchingolo et al. and others reported that osseodensification can be used to improve bone quality of the alveolar ridge and primary implant stability, e.g., via increased insertion torques [33,34]. Analogously, Elias et al. reported that parameters such as implant mode and size and surgical technique can influence primary implant stability. It is even known that tapered implants present higher insertion torques compared to cylindrical implant systems [35]. With respect to osseointegration, Cochrane et al. performed a study analyzing the influence of a mineral-organic adhesive on dental implants in over-dimensioned bony defects. They were able to show that the adhesive caused sufficient initial and later-implant stability. This might be based on a stepwise degeneration of the adhesive followed by sufficient osseointegration [36]. Other innovations focus on nest-like nanofiber structures to modify titanium implant surfaces and have been reported to improve osseointegration in animal models [37]. With respect to clinical conditions with a reduced bone turnover, such as osteoporosis, zinc- and strontium-modified implant surfaces showed an improved osseointegration in vivo [38]. Another promising option seems to be crosslinking of type I collagen on titanium implant surfaces. In particular, using gamma-rays induced an improved new bone area and bone implant contact compared to the controls [39].

Hydrophilicity is defined as an implant surface with a water contact angle <90°. Ultrahydrophilicity refers to implant surfaces with a water contact angle <10° which is associated with an increased protein formation responsible for cell attachment and migration on implant surfaces and an enhanced osteoblast differentiation, resulting in an improved and accelerated osseointegration [21,26,40]. Next, a positive influence of hydrophilicity on platelet activation and cytokine release, an upregulation of proangiogenic genes, and an enlarged macrophage activity was reported [41,42,43,44]. There is even an influence in the protein composition of salivary pellicles [45]. Consequently, an increased hydrophilicity might accelerate and improve the initial phase of dental implant osseointegration. Therefore, this study compared the osseointegration of an acid-etched, ultra-hydrophilic, micro-, and nanostructured implant surface compared to non-ultra-hydrophilic microstructured as well as non-ultrahydrophilic micro- and nanostructured implant surfaces in vivo.

## 2. Materials and Methods

### 2.1. Implants

Three different implants were tested. Common characteristics of the implants were a length of 11 mm, 3.3 mm diameter, a machined implant collar of 0.4 mm, a tapered geometry, and a flute design. First, Promote plus (Camlog^®^ screw line; Camlog Vertriebs GmbH, Wimsheim, Germany), characterized by a sandblasted (sb), acid-etched (ae), micro-structured (ms) and non-ultrahydrophilic (nuh) surface (contact-angle about 90°), referred to as SA (sandblasted, acid-etched), was used (*n* = 18). Next, two different implants (Logon^®^ OMF Bioactive Systems GmbH, Pforzheim, Germany) were tested. The first implant was exclusively produced for experimental use, characterized by a non-sandblasted (nsb), acid-etched (ae), micro- and nanostructured (ms, ns) and non-ultrahydrophilic (nuh) surface (contact-angle 90°), referred to as AN (acid-etched, nano-structured; *n* = 18). The second implant provides a nsb, ae, ms, ns, and uh surface (contact angle about 0°), referred to as ANU (acid-etched, nano-structured, ultra-hydrophilic; *n* = 18). Preservation of the ultrahydrophilicity is achieved by an innovative salt-coating which furthermore allows dry-packaging. Briefly, these ultra-hydrophilic implants were produced in a three-step process. Etching at elevated temperature led to an ultra-hydrophilic micro-structured surface. A nanostructure was generated by a proprietary process. Hydrophilicity was preserved in dry state by an exsiccation layer of salt.

### 2.2. Animals

All animal experiments were approved by the local ethical committee of the State University of Medicine and Pharmacy “N. Testemitanu”, Chisinau, Moldova and were carried out in accordance with the ARRIVE guidelines, in accordance with the U.K. Animals (Scientific Procedures) Act, 1986 and associated guidelines, EU Directive 2010/63/EU for animal experiments, and the National Institutes of Health guide for the care and use of Laboratory animals (NIH Publications No. 8023, revised 1978). In total, 27 female New Zealand rabbits, 6–8 months of age and each weighing between 4000 to 4500 g, were used. Each animal was housed in a single cage with a light-dark-cycle of 12 h and an unlimited food and water supply. The holding area had a mean temperature of 20–22 °C, a mean humidity of 55% and a ventilation of 18–20 times/h. The ground of the cages was filled with wood shaves. After surgery, the ground of the cages was filled with cellulose paper to avoid wound contaminations. The observation period started one week before surgery.

### 2.3. Surgery

First, 54 implants were randomly divided into 3 experimental groups, SA, AN and ANU, (*n* = 18) each. Surgery was performed under general anesthesia by intramuscular (i.m.) injection of ketamine (35 mg/kg bodyweight) and xylazine (5 mg/kg bodyweight). Perioperatively, vital parameters of the animals were controlled by a veterinarian. Before surgery, the proximal tibiae were shaved, epilated, and disinfected with 2% chlorhexidine solution, followed by an injection of local anesthetics (4% articaine with epinephrine 1:100.000; Septodont GmbH, Niederkassel, Germany). A vertical incision with a surgical blade was conducted up to the level of the periosteum. Next, the periosteum was prepared aside until the medial part of the tibia was exposed. Then, the preparation of the implant bed was started according to the manufacturers’ protocols, ending the preparation with an 3.3 mm diameter drill under continuous water cooling (0.9% saline solution). Implants were inserted with a final torque of 35 Ncm. Wound closure was performed in layers using 4-0 Vicryl (Ethicon GmbH, Norderstedt, Germany). Postoperatively, animals received antibiotics and analgesics: sol. Cefuroxime (Zinnat^®^, GlaxoSmithKline plc, Brentford, United Kingdom, Limited 125 mg/5 mL) intravenously 18.75 mg/kg as a single shot only and sol. Carprofen (Rimadyl^®^ 20 mL, Zoetis Inc., Parsippany-Troy Hills Township, NJ, USA) 4 mg/kg intramuscular every 12 h for 3 days. The wounds were protected with povidone iodine ointment. Animals of each group (SA, AN, ANU) were further assigned into three experimental subgroups, respectively. Group 1 was observed for 1 week, group 2 for 2 weeks and group 3 for 4 weeks, *n* = 6 each.

### 2.4. Histology

Once the observation periods of 1, 2, and 4 weeks expired, animals were sacrificed by an overdose of pentobarbital (120 mg/kg bodyweight). The tibiae were removed and fixed in 4% formalin. Further preparations of the samples were performed at the Department of Oral and Maxillofacial Surgery, University Medical Centre Mainz, Germany. To obtain histological sections, a grinding and sawing technique for hard tissue was used as described before [46]. The samples were cut down using a water-cooled saw (EXAKT GmbH, Norderstedt, Germany) and placed in an ascending series of alcohol, before being embedded into the Technovit 9100 system (Kulzer GmbH, Hanau, Germany). Afterwards, histologic sections were cut, followed by grinding and polishing according to standard protocols. Sections were then stained with toluidine blue for further evaluation according to standard protocols. Briefly, after 10 min in 0.1% formic acid, samples were put into 30% hydrogen peroxide for 20 min. Then, samples were put into a toluidine blue solution for 15 min with a subsequent drying and cleaning with a combination of acetone and alcohol (1:1). The cover glass was finally mounted with glycol methacrylate (Technovit 7210 VLC; Morphisto GmbH, Offenbach am Main, Germany) [47].

### 2.5. Histomorphometry

Histologic sections were digitalized with a light microscope (Biorevo BZ-9000; Keyence GmbH, Neu-Isenburg, Germany) at 1–10-fold magnification. The images were analyzed histomorphometrically with the freeware IMAGEJ for different parameters.

First, percentage of bone–implant contact in the cortical bone (cBIC, %) was analyzed. cBIC was separated into a “combined” cBIC, which was calculated by measuring the implant surface in contact to the bone divided by the total implant surface in the cortical bone area, and the new cBIC, which was calculated by measuring the implant surface in contact to unmineralized bone divided by the total implant surface in the cortical bone area. Both results were then multiplied by 100%. Further, percentage of bone-implant contact in the spongious bone (sBIC, %) was evaluated. sBIC was calculated by measuring the implant surface in contact to the spongious bone divided by the total implant surface in the spongious bone and then multiplied by 100%. Second, volume of bone within the screw thread with the highest amount of new-formed bone (BChI, %) was investigated. BChI was calculated by measuring the area of bone in the screw thread divided by the total area in the screw thread and then multiplied by 100%. Third, percentage of linear bone fill (PLF, % [48]) was analysed. PLF was calculated on the mesial and distal implant shoulder by measuring an 1.5 × 1.5 mm area. The bone-filled area inside the main area was divided by the total area and then multiplied by 100%.

### 2.6. Statistics

With a case number of *n* = 18 per implant and *n* = 6 per group, the present study had a similar if not higher number of samples when compared to similar studies [48,49]. For the statistical analysis, the software SPSS Statistics 20.0 for Macintosh (IBM, Armonk, NY, USA) was used. For cBIC, sBIC, and PLF, the left and the right implant shoulder were examined. For BChI, only the screw thread with the highest amount of bone was measured. For each of the parameters, mean values (M), standard deviations, and minimal and maximal values were generated. Since the experiments were exploratory, descriptive *p*-values of tests with a *p* ≤ 0.05 were termed as statistically significant.

## 3. Results

All animals were used for histological evaluation and data analysis with exception of 1 implant (SA, 4-week group) due to a lack of osseointegration.

### 3.1. Histological Evaluation after One Week

**Figure 1:**
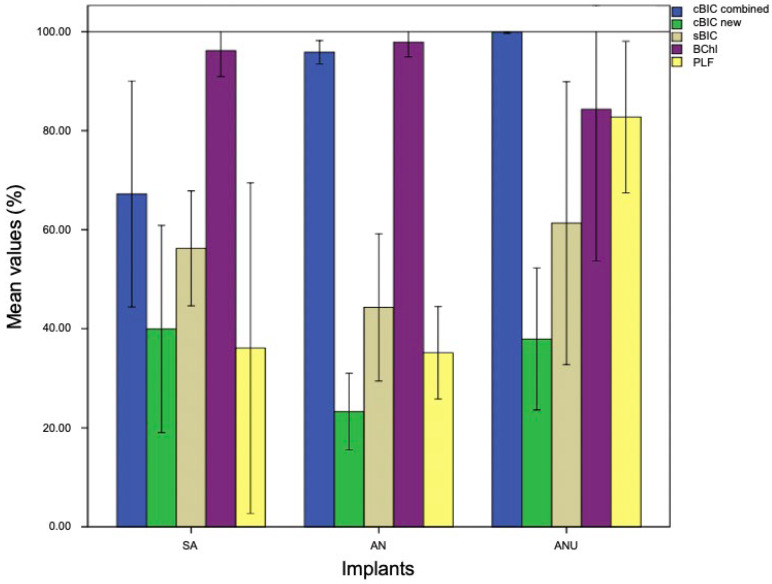
cBIC combined (blue), cBIC new (green), sBIC (sandy), BChI (purple), and PLF (yellow) of the tested implant surfaces SA, AN, and ANU in the rabbit tibia after 1 week. *x*-axis = tested implant surfaces, *y*-axis = mean values (%) ± SD.

**Figure 2:**
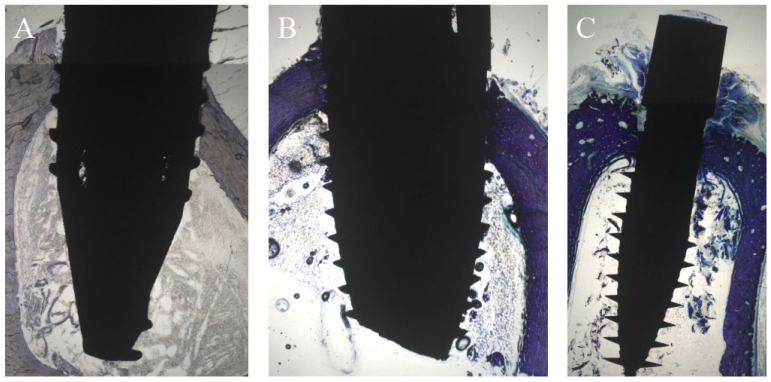
Histological analysis (toluidine-blue) of the tested implant surfaces. (**A**) SA, (**B**) AN (**C**) ANU in the rabbit tibia after 1 week.

After one week, SA (mean (M) 67.21%) demonstrated a significantly reduced cBIC when compared to AN (M 95.85%) and ANU (M 99.91%; *p* = 0.01 and *p* = 0.005). SA showed a slightly increased new cBIC when compared to AN and ANU but without any significant differences (*p* = 0.238 and *p* = 1). Concerning sBIC, ANU showed the highest values (M 61.33%), that was not significant in comparison to SA (*p* = 1) and AN (*p* = 0.367). With respect to BChI, AN demonstrated the highest bone ingrowth (M 97.91%) but without significant differences when compared to SA (*p* = 1) and ANU (*p* = 0.267). PLF for ANU (M 82.76%) was significantly increased when compared to SA (M 36.09%) and AN (M 35.14%) (*p* = 0.025 and *p* = 0.022; Figure 1). Figure 2 demonstrates histological slides of SA, AN, and ANU after 1 week.

Overall, the histological findings after one week demonstrated that the ultrahydrophilic implant surface demonstrated the highest bone-implant contact in the spongious bone compared to both other tested non-ultrahydrophilic implant surfaces. Next, the ultrahydrophilic implant surface showed a significantly increased percentage of linear bone fill compared to both other tested non-ultrahydrophilic implant surfaces. This overall indicates an accelerated and improved osseointegration of the ultrahydrophilic implant surface compared to the non-ultrahydrophilic surfaces within the early phase of osseointegration.

### 3.2. Histological Evaluation after Two Weeks

**Figure 3:**
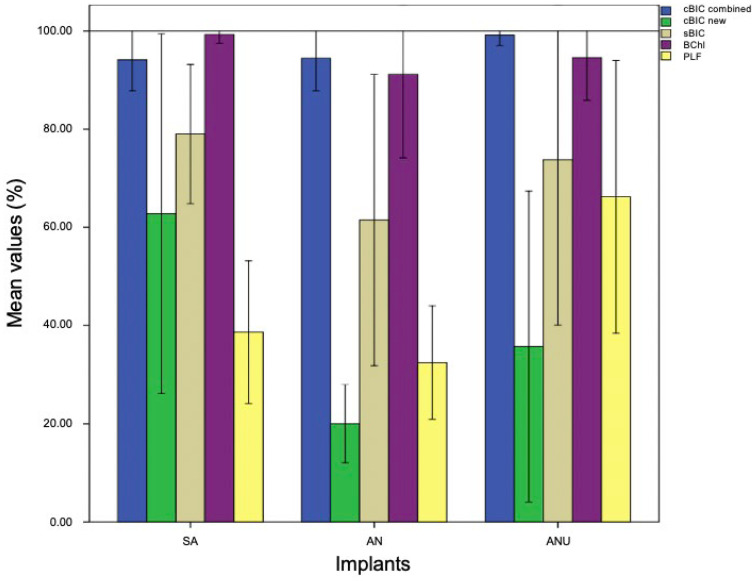
cBIC combined (blue), cBIC new (green), sBIC (sandy), BChI (purple), and PLF (yellow) of the tested implant surfaces SA, AN, and ANU in the rabbit tibia after 2 weeks. *x*-axis = tested implant surfaces, *y*-axis = mean values (%) ± SD.

**Figure 4:**
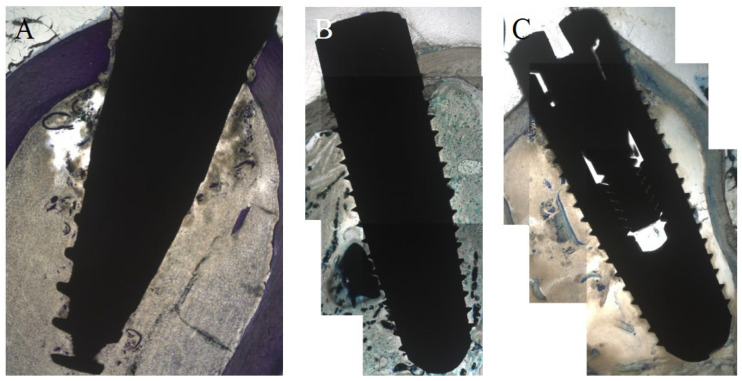
Histological analysis (toluidine-blue) of the tested implant surfaces (**A**) SA, (**B**) AN (**C**) ANU in the rabbit tibia after 2 weeks.

After two weeks, ANU demonstrated the highest cBIC (M 99.18%) that was not significant when compared to SA (*p* = 0.46) und AN (*p* = 0.535). Concerning the new cBIC, SA (M 62.81%) demonstrated the highest values that were significantly increased when compared to AN (M 20.02%) (*p* = 0.039). Compared to ANU (M 35.74%), there were no significant differences (*p* = 0.283). SA (M 79.03%) demonstrated an increased sBIC when compared to AN (M 61.50%) and ANU (M 73.81%) without significant differences (*p* = 0.925 and *p* = 1.0). SA (M 99.3%) showed a higher BChI than AN (M 91.19%) and ANU (M 94.60%) without significant differences (*p* = 0.949 and *p* = 1.0). ANU (M 66.24%) showed the highest PLF without any significant differences when compared to SA (*p* = 0.123) and AN (*p* = 0.57; Figure 3). Figure 4 is exemplarily demonstrating histological slides of SA, AN, and ANU after 2 weeks.

Overall, the histological findings after two weeks demonstrated that the ultrahydrophilic implant surface (ANU) showed the highest combined bone–implant contact compared to both other tested non-ultrahydrophilic implant surfaces. Next, the ultrahydrophilic implant surface demonstrated the highest percentage of linear bone fill compared to both other tested non-ultrahydrophilic implant surfaces.

### 3.3. Histological Evaluation after Four Weeks

**Figure 5:**
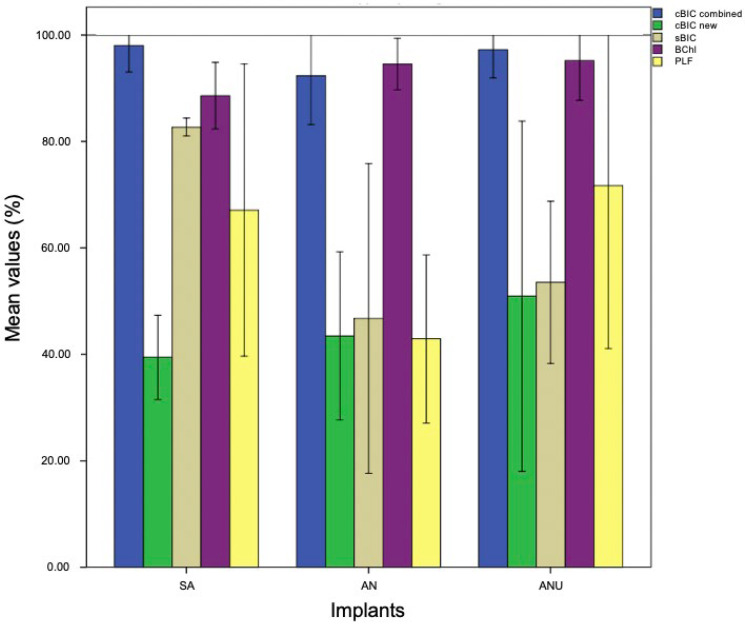
cBIC combined (blue), cBIC new (green), sBIC (sandy), BChI (purple), and PLF (yellow) of the tested implant surfaces SA, AN, ANU in the rabbit tibia after 4 weeks. *x*-axis = tested implant surfaces, *y*-axis = mean values (%) ± SD.

**Figure 6:**
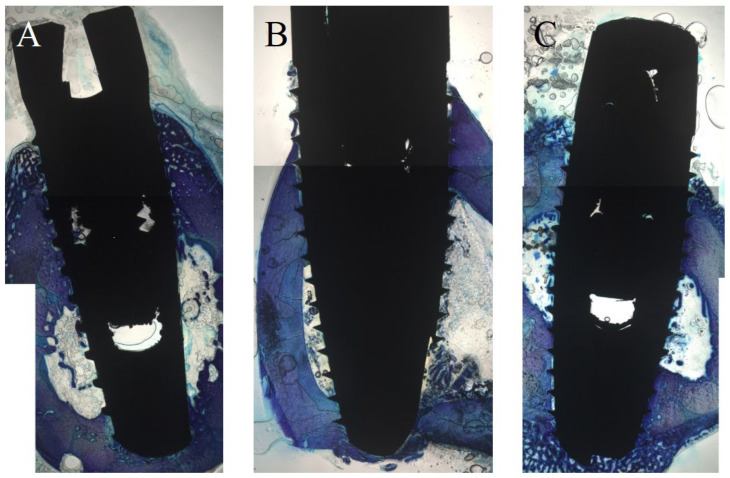
Histological analysis (toluidine-blue) of the tested implant surfaces. (**A**) SA, (**B**) AN, (**C**) ANU in the rabbit tibia after 4 weeks.

After four weeks, SA (M 98.05%) demonstrated the highest cBIC measurement. AN (M 92.35%) and ANU (M 97.25%) showed decreased cBIC values without any significant differences to SA (*p* = 0.675 and *p* = 1.0). With respect to the new cBIC, ANU (M 50.94%) had the highest values, without significant differences when compared to SA (*p* = 1) and AN (*p* = 1). SA (M 82.71%) demonstrated the highest sBIC but also without statistical significances when compared to AN (*p* = 0.06) and ANU (*p* = 0.261). ANU (M 95.21%) showed increased BChI values when compared to SA (M 88.62%) and AN (M 94.56%) without statistical differences (*p* = 0.277 and *p* = 1.0). ANU (M 71.69%) had the highest PLF values without significant differences when compared to SA (*p* = 1) and AN (*p* = 0.304; Figure 5). Figure 6 demonstrates histological slides of SA, AN and ANU after 4 weeks.

Overall, the histological findings after four weeks demonstrated that the ultrahydrophilic implant surface (ANU) demonstrated the highest new combined bone-implant contact compared to the other tested non-ultrahydrophilic implant surfaces. The ultrahydrophilic implant surface further showed an increased bone chamber ingrowth compared to the other tested non-ultrahydrophilic implant surfaces. Further, the ultrahydrophilic implant surface showed the highest linear bone fill compared to the other tested surfaces.

## 4. Discussion

Implant insertion is initially associated with bone damage. This results in a release of different growth factors, such as BMP-2, proteins, and stem cells to induce bone remodeling and new bone formation around the implant [50,51]. Next, to get a sufficient BIC, undifferentiated mesenchymal stem cells must migrate through the surrounding blood clot, adhere on the implant surface, proliferate and differentiate to mature cells [52]. Different studies have pointed out that hydrophilic implant surfaces enhance cell migration and adhesion onto the implant surface. This results in an overall accelerated and improved osseointegration [53,54]. In this context, some hydrophilic implants use wet-packaging in isotonic sodium chloride solution to keep the hydrophilicity [55]. This means that wet-packed implants lose the hydrophilicity faster when they are taken out of the package and get in contact with the air. Therefore, ANU seems to be a promising implant surface, since packing in sodium chloride is not necessary and hydrophilicity remains unchanged after unpacking. In ANU, hydrophilicity is preserved in dry state by an exsiccation layer of salt. This difference could be a significant clinical benefit. For this study, the rabbit model, which is a commonly used animal model for implant surface testing, was used [48,49]. The model is characterized by a short life cycle and a high bone-turnover rate among rodents [56,57]. In contrast to the current study, other studies tend to insert more than just one implant per tibia in the rabbit model which might lead to an overload with worse results [58]. But, even when placing one implant only, the tibia model in rabbits can simulate oral implant placement, only with some limitations. Substantial differences are varying amounts and thickness of cortical and spongious bone of the rabbit tibia compared to the human jaw as well as different biomechanical properties [59]. Alternatively, the sheep model is widely established, having even been used to test hydrophilic implants, demonstrating the benefit of hydrophilic surfaces for osseointegration [60,61]. According to the results, AN and ANU revealed nearly identical results for cBIC (M 95.85% vs. M 99.91%) after one week. Values for SA were significantly reduced. This could be explained by the existence of a nanostructure on AN and ANU implant surfaces which is missing on SA implant surfaces. Hydrophilicity does not seem to play a crucial role for cBIC. While cBIC, sBIC, and BChI are frequently used parameters, PLF is not so routinely used. It describes the supracrestal, subperiosteal newly formed bone which is widening the cortical zone. This results in a higher stability and promotes an uncomplicated healing process [49]. PLF for ANU (M 82.76%) was significantly increased when compared to SA (M 36.09%) and AN (M 35.14%) after one week. This could be explained by hydrophilicity, a property which is only present on ANU implants.

However, varying measuring methods have a direct impact onto the results. For example, other studies measured cBIC exclusively in threads while in this study the measurement started above the highest thread as long as it laid in the cortical zone of the bone [62] or even together with the sBIC as total BIC [63]. Another example is the measurement of BChI. Other studies measured all threads and determined a mean value while the presented study only measured the best thread in cortical zone [64]. Otherwise, measuring the best thread in the cortical zone is of significant clinical relevance since this area, including the peri-implant marginal bone, is an important starting point for marginal bone loss that might cause long-term side effects, such as periimplantitis [65]. While those differences might limit the comparability to other studies, those types of differences could be applied to nearly every study. Nevertheless, the results within this study are comparable with each other. While the results for ANU were already very sufficient after 1 week, ANU remained relatively unchanged until week 4, whereas SA and AN were closing the gap afterwards. In this study, cBIC was divided into combined and new cBIC while other studies tend to measure the combined cBIC only [64] or a total BIC combining cBIC and sBIC [63]. By measuring both combined and new cBIC, it is possible to observe the process of bone healing in a better way. Since new-formed unmineralized bone is going to become mineralized, decreasing new cBIC values and increasing combined cBIC values might display the healing process [66]. In the present study, effects of the nanostructure were exclusively seen for cBIC and of ultrahydrophilicity for PLF. This could be a result of the used 1- and 2-dimensional measuring techniques. Alternatively, 3-dimensional measuring techniques, such as micro-CT or synchrotron scans, could be used. Next, periosteal effect could explain these observations. In this context, it was demonstrated that periosteal stripping during surgical intervention significantly reduces cortical bone perfusion, by nearly 20% [67]. It could be possible that there is an unknown effect decreasing perfusion on cBIC and PLF in combination with the used surfaces. With respect to the potential effects of nanostructured implant surfaces, Jennissen designed a model regarding bone formation on nanostructured, hyperhydrophilic titanium surfaces and demonstrated significantly increased osteoid volume and bone and osteoid ingrowth after 4, 8, and 12 weeks compared to non-nanostructured surfaces. According to Jennissen, this could be based on a sufficient macrophage stimulation and consequently BMP-2 production by nanostructured surfaces [68]. Next, Vercellino et al. demonstrated that nanostructured titanium surfaces can support bone marrow stem cell differentiation [69]. Clinical relevance of hydrophilic implants, in the context of an accelerated and improved osseointegration, persists for immediate implant placement and loading as well as in patients with a reduced bone turnover. A reduced marginal bone loss can be seen as a further advantage, which has been demonstrated for hydrophilic surfaces in different studies [70,71]. This can additionally be associated with a decreased frequency of side effects, such as periimplantitis. In contrast, it was reported that hydrophilic surfaces might present an increased roughness. This might lead to more titanium particles released into the bone bed during implant placement as a possible reason for periimplantitis development [72]. To summarize, the ultra-hydrophilic and nano-structured implant surface showed superior results in the initial phase of osseointegration. Hence, it might be interesting to compare this implant surface with other ultrahydrophilic implants and further surface coatings, such as calcium phosphate and hydroxyapatite, in terms of future research. With respect to surface coatings and modifications, there is evidence that modified titanium surfaces, such as dual acid etched surfaces, can positively influence cell behavior of periodontal ligament stem cells and extracellular matrix formation [73]. Even surface modifications using phosphonic acid and a combination with BMP-2 showed beneficial influences on different cell lines, such as fibroblasts and mesenchymal stem cells [74]. Next, Al_2_O_3_ was described as a feasible surface modification for titanium implant surfaces presenting a positive influence on osteocytes and fibroblasts in vitro [75].

As a possible limitation of this study, animal models can only imitate the human bone turnover in restricted and inaccurate fashion, e.g., cause of an accelerated bone turnover. This can be critical especially with a focus on long-term results, e.g., for dental implant osseointegration. Nevertheless, the rabbit model is an established feature and frequently used in implant studies. For detailed information concerning the pros and cons of the rabbit model in dental implantology, see following reference [76]. Therefore, the ultrahydrophilic and nanostructured dental implant surfaces used in this study have to be evaluated in further clinical trials.

Overall, there exists a high count of surface modifications that are reported in the literature. The clinical challenge is the transfer of these findings into clinical application and further with long-term clinical results. We think that ultrahydrophilic titanium implant surfaces could represent a very promising option, since manufacturing is simple and the surface modification is simple and long-time stable.

## 5. Conclusions

An accelerated and enhanced initial osseointegration of ultrahydrophilic and nano-structured dental implants was found after 1 week that continued by trend after 2 weeks. After 4 weeks, no differences were detected between ultrahydrophilic and non-ultrahydrophilic dental implants. The exact mechanisms remain unclear and must be evaluated in further studies. Ultrahydrophilic dental implants might be a promising option in critical-sized alveolar defects and in cases with an affected and reduced bone turnover, such as in patients with radiotherapy of the head and neck area or receiving treatment with antiresorptive agents (e.g., bisphosphonates). Further relevant aspects of ultrahydrophilic and nano-structured dental implants might influence the long-term outcome including the implant survival and success rates and the development of implant-related complications, such as periimplantitis.

## Data Availability

Not applicable.

## Abbreviations

ae	acid-etched
uh	ultrahydrophilic
nuh	non-ultrahydrophilic
ms	microstructured
ns	nanostructured
sb	sandblasted
nsb	non-sandblasted
SA	sandblasted, acid-etched
AN	acid-etched, nanostructured
ANU	acid-etched, nanostructured, ultrahydrophilic
BIC	bone-implant contact
BChI	bone chamber ingrowth
PLF	percentage of linear bone fill
BMP-2	bone morphogenic protein
